# Prioritising research funding for cardiovascular disease and diabetes in Australia

**DOI:** 10.1057/s41271-023-00441-6

**Published:** 2023-11-28

**Authors:** Emily A. C. Grundy, Lauren E. Kelly, Erica Kneipp, Lucy Clynes, Alexander K. Saeri, Peter Bragge

**Affiliations:** 1 BehaviourWorks Australia, Monash Sustainable Development Institute, Monash University, 8 Scenic Blvd, Clayton, Melbourne, VIC 3800 Australia; 2 MTPConnect, Melbourne, Australia; 3 Australian National University, Canberra, Australia; 4 Research Australia, Canberra, Australia

**Keywords:** Research prioritisation, Co-design, Diabetes, Cardiovascular disease, Research strategy, Funding optimisation

## Abstract

The Targeted Translation Research Accelerator program was created to address cardiovascular disease and diabetes in Australia. To maximise the impact of the considerable investment in this program, a structured prioritisation project was undertaken to determine the highest priority health and medical unmet needs in cardiovascular disease and diabetes. The project was led by Monash University’s Behaviour Works Australia in collaboration with Australian National University, Research Australia, and MTPConnect. We conducted an online survey with 318 experts and community representatives to generate a ‘long list’ of unmet needs for (1) cardiovascular disease; (2) diabetes; and (3) interactions in the pathogenesis of Type 1 diabetes, Type 2 diabetes, and cardiovascular disease. We then convened roundtables of clinical, research, and community leaders to discuss survey results. They prioritised unmet needs against six predefined criteria then discussed results. We present the final priority areas for funding. We demonstrate how a feasible, reproducible, and collaborative prioritisation methodology can be used when designing research funding programs. Such approaches can ensure that funding is directed towards projects that are valuable to the community and reflective of expert opinion.

**Supplementary Information:**

The online version contains supplementary material available at 10.1057/s41271-023-00441-6.

## Key messages

- Feasible, reproducible, collaborative, and transparent prioritisation approaches can guide research funding and associated policy agendas.
- This research identifies high priority areas for research funding in cardiovascular disease and diabetes.

## Introduction

Cardiovascular disease (CVD) and diabetes pose a substantial global disease burden, representing over 25% of early mortality and 20% of disability adjusted life years lost [1]. In Australia, the burden of these diseases affects millions of people [2, 3]—causing direct suffering, as well as lost social and economic value. Therefore, the Australian Government has prioritised research into these diseases [4, 5].

In 2020, the Australian Government allocated AUD 47 million to fund CVD and diabetes research through the Medical Research Future Fund (MRFF), in recognition of the considerable health burden experienced by the Australian people and to support the goal of social and economic health [6]. MTPConnect, Australia’s medical technology, biotechnology, and pharmaceutical Growth Centre, was assigned responsibility by the Australian Government to design and deliver this major MRFF program of funding, hereafter referred to as the Targeted Translation Research Accelerator (TTRA) program for diabetes and CVD. The aim of the TTRA program was to improve the prevention, diagnosis, treatment, and management of CVD and diabetes and their associated complications in Australia. To achieve this aim, MTPConnect sought to establish two Research Centres; fund individual research projects through a series of funding rounds; and promote implementation and commercial translation of interventions.

**Fig. 1:**
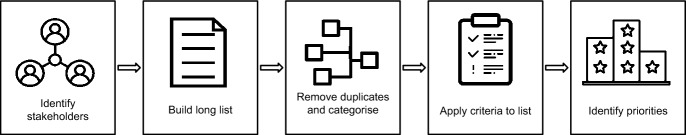
Summary of steps involved in a prioritisation process

As part of its delivery of the TTRA program, MTPConnect sought to ensure that its research activities delivered impact, built upon existing initiatives, and filled gaps in the sector. To meet these requirements, MTPConnect embedded a structured prioritisation process throughout its delivery of the TTRA program. Structured approaches to identifying and prioritising options involve (1) identifying a broad community of invested stakeholders; (2) building a ‘long list’ of possible options through engagement with this community (for example, using a survey) or reviewing existing research; (3) removing duplicates from and categorising the ‘long list’ of options; (4) applying predetermined, explicit prioritisation criteria to the deduplicated ‘long list’ in order to; (5) identify the highest priority options [7, 8]. Figure 1 provides a summary of this process.

Use of a structured prioritisation approach can aid organisations in scoping, defining, and understanding where investments in health research are best directed. Appropriately engaging stakeholders and applying evidence-based co-design principles (such as allocating sufficient time and resources, managing expectations, validating and empowering participants) can also build legitimacy [9]. A structured prioritisation approach does have limitations; in that it cannot consider every possible option [10] nor consult every conceivable stakeholder influenced by the decision [11]. It is essential, however, for optimising the use of scarce resources such as time, expertise, and funding [10, 11], and enables funding organisations to explicitly and transparently report on how they have identified priority areas.

This study applied a structured prioritisation approach to identify priority unmet needs—defined as medical or health-related issues not adequately addressed by current interventions—to inform research funding rounds conducted by MTPConnect as part of its TTRA program delivery. Specifically, the process aimed to identify the top three unmet needs for each of the three program focus areas: (1) Complications associated with CVD; (2) Complications associated with diabetes; and (3) Interactions in the pathogenesis of Type 1 diabetes, Type 2 diabetes, and CVD. Although the focus of this prioritisation process was on complications and disease interactions, we did not neglect the concept of prevention: responses to the final research funding rounds could encompass various approaches, including prevention, diagnosis, treatment, or management strategies.

## Methods

We designed the study using a mixed method application of structured prioritisation principles that have been applied in numerous research policy settings [7, 8, 12–15]. This step-by-step process is based on multi-criteria decision analysis [16], and emphasises a participatory and consultative approach. The project comprised three major stages:

1. Determining the prioritisation process, goals, and scope;
2. A national survey of experts and community representatives, to create a ‘long list’ of potential unmet needs; and
3. Prioritisation roundtables where experts and community representatives applied explicit prioritisation criteria to the deduplicated ‘long lists’ to determine the highest priority unmet needs for funding.

The research activities in this project received ethical approval by Monash University’s Human Research Ethics Committee (Project Number: 26586).

### Stage 1: Determining the prioritisation process, goals, and scope

The research team (EG, EK, LC, AS, PB), in collaboration with MTPConnect (represented by LK), determined the goals and scope of the prioritisation process. This was then endorsed by the independent TTRA Expert Advisory Board. This was especially important because the priority unmet needs would form the basis of calls for research funding, and research activities supported by the funding needed to achieve the objectives of the TTRA program. Table 1 presents the resulting prioritisation parameters.

**Table 1:** Prioritisation scoping questions and decisions

Scope question	Decision
What is being prioritised?	Unmet needs in (1) Cardiovascular disease; (2) Diabetes; and (3) The interaction between cardiovascular disease and diabetes
How will stakeholders generate options for the ‘long list’?	People with expertise in cardiovascular disease and/or diabetes will be asked to identify unmet needs, in the form of research topics, related to cardiovascular disease and/or diabetes People with knowledge of lived experience (i.e. community representatives) will be asked to describe the complications arising from cardiovascular disease and/or diabetes that they believe need to be addressed
What criteria will be used to prioritise the options?	Six criteria (outlined in Table 2) will be used to prioritise the deduplicated ‘long list’ of unmet needs
What is the final number of priorities required?	Three priorities per focus area

**Table 2:** Elicitation of unmet needs for cardiovascular disease, diabetes, and their interactions in the national survey (experts vs community representatives)

Focus area	Audience	
	Community representatives	Experts
Diabetes	List at least two complications you would like addressed relating to diabetes, and the impact that those complications have	List at least two research topics that address a disease-related complication associated with diabetes
Cardiovascular disease	List at least two complications you would like addressed relating to cardiovascular disease cardiovascular disease, and the impact that those complications have	List at least two research topics that address a disease-related complication associated with cardiovascular disease
Interactions	List at least two complications you would like addressed relating to having both diabetes and cardiovascular disease, and the impact that those complications have	List at least two research topics in the area of better understanding and treating interactions in the pathogenesis of Type 1 diabetes, Type 2 diabetes, and cardiovascular disease

### Stage 2: National survey

We developed a survey of Australian adults with expertise or knowledge of the lived experience of CVD and/or diabetes to generate a ‘long list' of unmet needs. We chose this method to maximise national reach and ensure that each respondent could respond anonymously. The target audience of the survey were (1) adults with expertise in CVD and/or diabetes, including people who specialised in treatment, research, or management; and (2) adults who lived with, or cared for, people with, CVD and/or diabetes (henceforth referred to as community representatives).

We co-designed the survey with methodology and recruitment experts, MTPConnect, and the TTRA Expert Advisory Board to ensure it would be accessible to the target audience and align with the project goals and scope (see Stage 1). The full survey is presented in Supplementary Materials (Supplementary File 1: Survey). We collected data on whether participants had expertise in CVD and/or diabetes (e.g., researchers, clinicians) or had knowledge of the lived experience of these diseases, as well as demographic information. The survey included questions intended to elicit unmet needs for CVD or diabetes, or both, from participants. We used different wording for experts and for community representatives to maximise accessibility. Table 2 summarises how we elicited unmet needs for each audience group and focus area. See Supplemental Materials (Supplementary Files 2–4) for the relevant surveys.

#### Survey participants and recruitment

MTPConnect and Research Australia, the national peak body for Australian health and medical research, distributed the survey to approximately 500 individuals and organisations. The distribution list included Australian health and medical researchers, clinicians, health professionals, policymakers in state and national health departments, and national and international private organisations with interests in CVD and/or diabetes. The research team asked not-for-profit organisations working in CVD and/or diabetes to distribute the survey through their community networks. We encouraged explicit efforts to reach community networks, given that their involvement improves research outcomes by ensuring relevance to community needs [7, 17]. The team sent a follow-up reminder a week after the initial survey invitation and held the survey open for two weeks.

#### Analysis of unmet needs

We conducted inductive thematic analysis to organise the unmet needs identified in the survey. The inductively derived coding framework had two levels: Level 1 codes for the body system (for example, Cardiac), and Level 2 codes for the specific body part or issue within that system (Cardiomyopathy or heart failure). Two researchers independently coded 100 unmet needs to develop an initial coding framework. The research team discussed discrepancies, then refined the coding framework. One researcher coded the remaining unmet needs for each focus area (CVD, diabetes, or interactions in the pathogenesis of Type 1 diabetes, Type 2 diabetes, and cardiovascular disease). The research team met regularly throughout the coding process to maximise consistency of the approach and to further refine the coding framework.

#### Finalising the lists of identified priorities

Once all unmet needs had been coded, we cleaned the dataset and removed duplicates. We removed incomplete survey responses and responses that could not be interpreted as unmet needs (such as generic statements like ‘use technology to improve diabetes management’). Removal of duplicates involved combining unmet needs that were very similar, but which used different wording, into a single coded unmet need (for example, the responses ‘Islet transplantation to address diabetic retinopathy resulting in less vision loss’ and ‘Early treatments for diabetic retinopathy to prevent progression to vision loss’ were combined into the unmet need ‘Eye, Retinopathy’). This meant we gave equal weight to all unmet needs generated in subsequent prioritisation, because the final deduplicated ‘long list’ did not convey how many survey responses had mapped to each coded unmet need.

### Stage 3: Prioritisation roundtables

We conducted three roundtables to apply pre-specified and explicit prioritisation criteria to the deduplicated ‘long lists’—to determine the highest priority unmet needs. Each roundtable addressed one of the three focal areas: (1) Complications associated with diabetes; (2) Complications associated with CVD; or (3) Interactions in the pathogenesis of Type 1 diabetes, Type 2 diabetes, and CVD.

#### Prioritisation criteria

The TTRA Expert Advisory Board and MTPConnect developed the prioritisation criteria (presented in Table 3), drawing upon criteria used in previous projects [12].

**Table 3:** Prioritisation criteria for unmet needs in cardiovascular disease and diabetes

Name	Description	Weight
Clinical impact	Effect of the medical or health-related unmet need	2
Quality of life	Impact on patient quality of life	2
Consumer expectations	Consumer needs not adequately addressed	1
Diversity/regional, rural, remote impacts	Greatest inequities for underserved populations	1
Commercial potential	Profitability based on current or predicted market	1
Productivity outcomes	Economic benefit in addressing the unmet need (e.g. increased productivity or participation)	1

Up to ‘‘Consumers’ refer to adults who live with, or care for people with, CVD or diabetes, or both (i.e., community representatives)’. Project personnel refer to consumer participants as ‘community representatives’

The TTRA Expert Advisory Board decided that clinical impact and quality of life should be weighted as double the value of other criteria, because a key objective of the TTRA program funding is to improve clinical practice to reduce the burden of CVD and diabetes.

#### Roundtable recruitment and procedure

Following consultation with the TTRA Expert Advisory Board, we approached a national and diverse list of organisations to nominate experienced individuals to participate in the roundtables. We took care to include organisations which would bring diverse yet complementary perspectives including clinical, research, health professional, health administration, Aboriginal and Torres Strait Islander health, community, industry, and investor. Following their nomination, we sent the individual participants an explanatory statement and asked them to provide consent prior to attending the roundtable.

We conducted the roundtables online, each for 2.5 hours in length. See Supplementary Files 2, 3, and 4 for roundtable agendas, briefing information for participants, and the deduplicated 'long list' of unmet needs. We followed the Chatham House Rule [18] meaning that we did not disclose the identity of participants following the roundtable nor attribute statements publicly. We provided participants with an overview of the project and time to read the deduplicated 'long list' of unmet needs. Participants then considered each prioritisation criterion in turn, and anonymously voted for their top three unmet needs according to that criterion, using an interactive polling platform. We calculated the votes for each unmet need across all criteria (with the clinical impact and quality of life criteria weighted at double). Participants discussed the top five prioritised unmet needs in facilitated groups. Guiding questions encouraged participants to discuss their initial reactions to the results, their knowledge of the current state of the science, and how the impact of the TTRA funding could be maximised.

The TTRA Expert Advisory Board used the research report summarising the voting results and key themes to finalise the top three priorities in each focus area that formed the basis for research funding calls.

## Results

Table 4 presents the overall flow of data, the number of unmet needs initially suggested in the survey, the number of deduplicated unmet needs after coding, the top five priorities emerging from the roundtables, and the final TTRA funding calls. Across the focus areas, on average, survey respondents were split evenly between males (49%) and females (51%), three quarters were experts (75%), and one quarter community representatives (25%). The work of most experts focused primarily on major cities (65%) or had no geographical focus (21%). See Supplementary Materials (Supplementary File 5: Survey Demographics) for a more detailed demographic breakdown.

**Table 4:** Unmet needs for the three TTRA focus areas at each stage of the prioritisation process

Stage	Cardiovascular disease	Diabetes	Interactions
National Survey	319 unmet needs	351 unmet needs	242 unmet needs
Deduplicated ‘long list’	19 unmet needs	24 unmet needs	29 unmet needs
Roundtable priorities	1. Cardiac, Coronary artery disease/Angina/Major adverse cardiovascular events 2. Cardiac, Cardiomyopathy/Heart failure 3. Cerebrovascular, Transient ischaemic attack & Stroke 4. Effective Practice/Organisation of Care 5. Cerebrovascular, Cognitive impairment/Dementia	1. Diabetic kidney disease 2. Mental health 3. Cardiac, cardiovascular disease/Coronary artery disease/Major adverse cardiovascular events 4. Glucose control, Hypo/Hyperglycaemia, Diabetic ketoacidosis 5. Diabetic foot	1. Mental health 2. Diabetic kidney disease 3. Cardiac, Cardiovascular disease 4. Primary prevention 5. Evidence-practice gaps
Funding calls	1. Coronary artery disease (including angina and major adverse cardiovascular events); 2. Cardiomyopathy/heart failure; 3. Transient ischaemic attack/stroke (ischaemic and haemorrhagic)	1. Diabetic kidney disease; 2. Peripheral neuropathy and diabetic foot syndrome; 3. Short-term complications of hypoglycaemia and/or hyperglycaemic hyperosmolar syndrome and ketoacidosis	1. Mental health conditions; 2. Chronic kidney disease; 3. Cardiac and vascular complications

### Roundtable results and funding calls

For each focus area, the following sections describe (1) the top five prioritised unmet needs; (2) a summary of participant reflections on the prioritised unmet needs; and (3) the final funding call.

#### Cardiovascular disease

For cardiovascular disease, the top five unmet needs are shown in Table 4. Overall, participants did not express surprise at the top five unmet needs and agreed that the major areas of need were coronary disease, cardiac failure, and stroke. Discussion touched on several areas outside of the top five priorities, including:

- The strong association between CVD and mental health. Participants emphasised that mental health challenges are often an undiagnosed part of stroke, masked by cognitive complications;
- Community representatives expressed concerns about access to specialist services and about how CVD affects the ability to exercise;
- How treatment can be improved in different populations.

Participants suggested that promising interventions should involve technical solutions and changes to health systems, and consider environmental and lifestyle associations with CVD complications. Although there was broad agreement with the prioritised unmet needs, participants emphasised that the results were likely influenced by who was present at the roundtable (e.g., their specific interests and experiences) and the use of a coding framework for classifying the unmet needs (which was used to code the survey data and, therefore, was the foundation for the long list of unmet needs).

The TTRA Expert Advisory Board considered the top five unmet needs, and the broad agreement voiced in the roundtable, and resolved to call for funding for the top three:

- Funding call 1: Coronary artery disease (including angina and Major adverse cardiovascular events);
- Funding call 2: Cardiomyopathy / heart failure;
- Funding call 3: Transient ischaemic attack / stroke (ischaemic and haemorrhagic).

#### Diabetes

The top five unmet needs that emerged from the diabetes roundtable voting are shown in Table 4. Participants regarded the top priority of diabetic kidney disease as very important. There was notable discussion around the second priority of mental health. Participants noted that it was a substantial issue for the community, especially when the chronic illness onset is earlier in life (i.e. childhood/early adulthood). Participants discussed the difficulty of untangling mental health from the other prioritised unmet needs. They expressed a lack of certainty about how the TTRA program should address mental health needs as a specific complication of diabetes when there are other national research funding schemes that support mental health research in general. As in the CVD roundtable, participants commented on the effect of categorisation choices (i.e. coding framework used to analyse survey data) and participant interest/experience. There was consensus that the neuropathy and diabetic foot categories should be combined, and that the absence of eye disease in the top five priorities may reflect the limited ophthalmological presence at the roundtable. Participants emphasised that inequality (in diagnosis and access to treatment) persisted in all top five unmet needs. Participants agreed that diabetic kidney disease, CVD, and diabetic foot unmet needs could be addressed through research into novel biologics, screening and monitoring, new medication models, and improving adherence. 

The TTRA Expert Advisory Board resolved to retain the top priority of diabetic kidney disease in the funding call, given the agreement with its importance. After merging neuropathy and diabetic foot, following advice from the roundtable discussion, this category rose to second priority. As mental health was the top priority in the Interactions roundtable (see below), the TTRA did not include it in the diabetes funding call to avoid funding duplication. Similarly, although CVD emerged as a leading unmet need, the TTRA considered it out of scope to avoid duplication with the CVD and interactions funding calls.

Consequently, the unmet need regarding glucose control rose to third priority. The TTRA Expert Advisory Board finalised the following funding calls:

- Funding call 1: Diabetic kidney disease;
- Funding call 2: Peripheral neuropathy and diabetic foot syndrome;
- Funding call 3: Short-term complications of hypoglycaemia and/or hyperglycaemic hyperosmolar syndrome and ketoacidosis.

#### Interactions in the pathogenesis of Type 1 diabetes, Type 2 diabetes, and Cardiovascular disease

The top five unmet needs that emerged from this roundtable voting are shown in Table 4. There was general agreement that the top five priorities were important. Mental health was extensively discussed, with a focus on its compatibility with the TTRA program, whether it is a complication of CVD and diabetes or a complication in the management of those conditions, and terminology (such as mental illness vs. mental health). Similarly, these roundtable members saw gaps between evidence and practice as important matters and questioned whether they were in scope for the TTRA. Topics of interest identified as important at the roundtable, but not within the top five priorities, included islet and b-cell regeneration, inflammation and its precursors, cardiomyopathy, atherosclerosis, and wound healing. Roundtable participants identified Australian research strengths relevant to this area as big data, genomics, and strong clinical partnerships in the cardiometabolic space, as well as clinical trials capability. Participants identified renal dysfunction and islet transplantation as potential focus areas, while suggesting that cardiac risk prediction was already well covered. Similar to the other two roundtables, participants in this one discussed the effect of ‘lumping and splitting’ (i.e. the effect of the coding framework on the creation of the long list, which involved making decisions on grouping concepts into one unmet need or as separate unmet needs) and how this may have influenced voting results. The TTRA Expert Advisory Board resolved to pursue the calls outlined in Table 4, as follows:

- Funding call 1: Mental health conditions in people living with at least two of the following: Type 1 diabetes, Type 2 diabetes and/or cardiovascular disease; or
- Funding call 2: Chronic kidney diseases in people living with at least two of the following: Type 1 diabetes, Type 2 diabetes and/or cardiovascular disease; or
- Funding call 3: Cardiac and vascular complications arising in people living with diabetes (Type 1 or Type 2) and cardiovascular; OR Cardiovascular disease in people living with Type 1 diabetes and insulin resistance (double diabetes).

## Discussion

This paper documents the use of a transparent, reproducible, consultative, collaborative, and data-driven process to determine expert and community priority areas for CVD and diabetes. An Australian national survey involving 318 participants generated a total of 912 unmet needs. The deduplicated ‘long lists’ of unmet needs were then prioritised and discussed at roundtables. The TTRA Expert Advisory Board, in consultation with MTPConnect, distilled the priorities into nine funding calls for research across the three CVD and diabetes focus areas.

The applied research approach described in this paper has two key strengths. First, it exemplifies how a transparent and inclusive method can be used in decision making for government research funding. We adapted the method from several previous prioritisation projects with varying units of prioritisation and prioritisation criteria [7, 8, 12–15]. The methods emphasise inclusivity, involving purposive recruitment of domain experts (such as researchers, clinicians, health professionals) and, crucially, people and groups affected by funded research (including carers, community organisations, people living with CVD and/or diabetes) in both identifying and prioritising unmet needs for funding. This approach addresses common concerns that decisions about what to fund are influenced by subjectivity and bias [11]. Although the TTRA Expert Advisory Board participated in setting project parameters, such as the target number of funding calls and prioritisation criteria, they had no role in data collection, analysis, nor synthesis. This helped reduce potential conflicts of interest. It also increased legitimacy of the process for participants by signalling that the funder wished to consult experts and the community to determine the areas for research funding offering greatest impact. Finally, we took care to mitigate potential participant conflicts of interest by (1) including all unique unmet needs in the long list, regardless of how often it was mentioned (meaning if a survey participant suggested something multiple times, it was only included once), and (2) ensuring all roundtable participants had equal say in the prioritisation voting process.

The second strength of this research approach is that it demonstrated the feasibility of embedding best-practice prioritisation processes in the development of research calls. The prioritisation process deployed for the TTRA program could be applied to other medical research initiatives and government programs. Although this project focused on Australia, the prioritisation approach could be reproduced internationally. Following an explicit and transparent method meant that we were able to conduct the process relatively quickly. The cost of the prioritisation project amounted to a fraction of the research funding allocated.

Project limitations also warrant mention. Firstly, the stakeholder engagement process could be improved. As it was not possible to consult widely on the prioritisation criteria in the scope of this project, the TTRA Expert Advisory Board determined them. This board did, however, include representatives from the clinical, research, and policy sectors, in addition to community representatives. Although the online survey used to collect unmet needs provided an easy and timely way to access many stakeholders, we did not reach certain expert and community populations adequately. Overall, community representatives made up a minority of the survey sample, and we lacked participants in or focused on rural and remote communities. Prioritisation roundtables also lacked broader representation of some communities most affected by CVD and diabetes, including Aboriginal and Torres Strait Islander communities. MTPConnect and the TTRA Expert Advisory Board recognised this gap and have addressed it in a subsequent prioritisation project.

Another challenge in this project was accounting for the nuances within the ‘long list’, while also seeking to reduce the unmet needs to a number that roundtables could feasibly assess and prioritise. The coding frameworks lacked sufficient clinical input, and some decisions about when to lump or split categories were made by research team members without extensive clinical expertise in the topic areas. Nonetheless, roundtable participants did have opportunities to discuss the ‘lumping and splitting’ of unmet needs. This underlines the importance of deliberating prioritisation findings rather than solely focusing on the numerical rankings. Future research could further optimise the categorisation or deduplication step of the prioritisation process. Drawing upon established taxonomies specific to the topic area, for example, could add rigour to data analysis.

This project is well-aligned with the Australian Government’s ambition to effectively engage the public in policy development and program design. It builds on existing community-focused activities required by legislation supporting the Medical Research Future Fund (MRFF), an AUD 20 billion Australian investment. Every two years, the Australian government invites public submissions to inform the priorities of the MRFF and ensure that community voices inform the global setting of MRFF priorities. The current project translates similar prioritisation principles to a more granular, operational level.

The Australian Public Service’s Framework for Engagement and Participation states that engaging with members of the public goes beyond disseminating policy decisions or submission-based consultations [19]. This project demonstrates how this advice can be actioned by incorporating other forms of community involvement. Community representatives contributed to the long-listing process, prioritising unmet needs, and in roundtable discussions. Such involvement brings the community one step closer to shaping research that is relevant for them.

## Conclusions

The impact of this research is twofold. First, the research findings had an immediate, visible, and non-trivial influence on the TTRA program funding calls, which totalled approximately AUD 25 million. Second, this project demonstrates how government can embed a prioritisation process in, and use it to help shape, funding decisions. The problem of ‘what to fund’ is not unique to the TTRA program, nor to Australia. This feasible, reproducible, collaborative, and transparent approach could be useful for other funding bodies and policy makers in setting future funding and policy agendas. When resources are limited, evidence-based prioritisation methods such as this one can be used to maximise impact.

### Supplementary Information

Below is the link to the electronic supplementary material.

Supplementary file1 (PDF 705 kb)

## Data Availability

The datasets used and/or analysed during the current study are available from the corresponding author on reasonable request.
