# Supplementary material for: Prioritising research funding for cardiovascular disease and diabetes in Australia
Source: J Public Health Policy. 2023 Nov 28;44(4):658–73. doi: 10.1057/s41271-023-00441-6 (PMC10709470; doi:10.1057/s41271-023-00441-6)
Supplement: Supplementary file 1 — Supplementary file1 (PDF 705 kb) [file 41271_2023_441_MOESM1_ESM.pdf]

Journal of Public Health Policy

Supplementary Materials

Prioritising Research Funding for Cardiovascular Disease and Diabetes

Emily A. C. Grundy[1], Lauren E. Kelly[2], Erica Kneipp[3], Lucy Clynes[4], Alexander K. Saeri[1], Peter Bragge[1]

1. BehaviourWorks Australia, Monash Sustainable Development Institute, Monash University, Melbourne, Australia
2. MTPConnect, Melbourne, Australia
3. Australian National University, Canberra, Australia
4. Research Australia, Canberra, Australia

Corresponding authors:

Emily Grundy: emily.grundy@monash.edu

Peter Bragge: peter.bragge@monash.edu

BehaviourWorks Australia, Monash Sustainable Development Institute, Monash University, 8 Scenic Blvd, Clayton, Victoria, 3800, Australia.

Running title:

Prioritising Research Funding

Supplementary File 1: Survey Instrument

Prioritising Research in Diabetes and Cardiovascular Disease

Welcome to the Prioritising Research in Diabetes and Cardiovascular Disease (D & CVD) survey. Before you continue, please read the important explanatory and consent information below in full before deciding whether or not to participate.

If you have already completed this survey, you are not eligible to participate again - please exit the survey.

What does the research involve?

You are invited to take part in a 10 – 15 minute survey which is being administered by MTPConnect and funded by the Medical Research Future Fund (MRFF). No pre-reading or other preparation is required to complete the survey. The survey is being sent to people with expertise and / or experience in D & CVD. This includes doctors, researchers, industry professionals, and consumers (e.g., someone who lives with one or both of these conditions, a carer or family member of someone who lives with one or both of these conditions, or a person working in an advocacy organisation).

The survey is part of a project which aims to gather and prioritise research topics in the fields of D & CVD. The Australian Government recently announced a $47 million investment in D & CVD research — the Targeted Translation Research Accelerator (TTRA) initiative. The TTRA is funded by the Medical Research Future Fund (MRFF) and will be operated by MTPConnect. Researchers will be able to apply for research funding in December 2020.

Initially, the TTRA will focus on three areas:

1. Preventing and managing complications associated with diabetes;
2. Preventing and managing complications associated with cardiovascular disease; and
3. Exploring interactions in the pathogenesis of Type 1 diabetes, Type 2 diabetes, and cardiovascular disease that may lead to new interventions for people with these conditions

If you do not have expertise in or experience with diabetes and / or cardiovascular diseases, you are not eligible to complete this survey - please exit the survey.

The findings of the survey will be anonymously collated and deduplicated to produce a list of all research topics identified by survey participants.

This list will inform a series of three 2-hour online roundtable discussions - one for each of the three TTRA focus areas listed above. The roundtables will be attended by approximately 25 people with expertise and / or experience in D & CVD. A professional facilitator will guide roundtable participants in a discussion of the list of research topics. The top three research topics will be prioritised at the roundtable based on the following pre-specific criteria set by the TTRA Expert Advisory Board:

- Clinical impact (weighted as twice as important as other criteria)
- Quality of life (weighted as twice as important as other criteria)
- Commercial potential
- Economic outcomes

- Diversity / regional, rural, remote impacts
- Consumer expectations

Why are you being asked to complete this survey?

You have been invited to participate in this survey based upon your expertise and / or experience in the field of D & CVD. People who specialise in either treating, researching, or managing D & CVD and people who live with either or both of these conditions are best placed to identify areas in which research is required. This survey seeks to gather a broad range of research topic ideas from researchers, clinicians, consumers, policymakers, industry, and other stakeholders in these areas.

Possible benefits and risks to participants

There should be no inconvenience or discomfort experienced by participants in this research, other than the amount of time to participate. The benefit of participation is that your input will contribute to guiding this substantial investment in D & CVD research.

Confidentiality & results

All information collected from you will remain confidential with only the researchers having access to the study information unless otherwise required by law. Upon completion of the research, a report will be provided to MTPConnect and the TTRA Expert Advisory Board in summary format so that no individual can be identified. The research findings may also be submitted for publication or used in conference presentations (also in summary format). MTPConnect provides funding through open calls to this sector. Because this survey is entirely voluntary and anonymous, it cannot be linked to any past, current, or potential future funding calls made by MTPConnect. Therefore, your participation in this survey cannot influence any research funding applications you may make to MTPConnect.

Storage of data

Upon completion of the project, data files will be held for a minimum of five years, secured on the Monash University network with restricted access, limited to the research staff only. The data may be retained and used by the research team for comparative purposes in the future.

Consent and withdrawal

To consent to participating in this research project, please tick the box below and proceed to the survey. You are free to withdraw from participation at any time prior to completing the survey. It will not be possible for you to withdraw participation after completing and submitting the survey, as from this point your contributions cannot be de-aggregated from those of others. There are no consequences for not participating in this research.

Complaints

If you have any concerns or complaints about the project, you can contact the Executive Officer of the Monash University Human Research Ethics Committee.

Project number: 26586

Executive Officer, Monash University Human Research Ethics Committee

(MUHREC) Room 111, Building 3e, Research Office, Monash University VIC 3800
Tel: +61 3 9905 2052
Email: muhrec@monash.edu
If you would like further information regarding any aspect of this project, you are encouraged to contact the investigator listed below. Thank you for your consideration of this project.

Associate Professor Peter Bragge
Lead Chief Investigator, TTRA Prioritisation project
BehaviourWorks Australia, Monash Sustainable Development Institute
Monash University, Melbourne
peter.bragge@monash.edu

- I DO consent to completing this 15-minute survey
- I DO NOT consent to completing this 10 - 15 minute survey

LOGIC - if CONSENT == "I DO NOT consent", exit survey and show

- Thank you for your consideration. You may now close this window as you have elected not to participate in the survey

SECTION A: Your expertise or experience with D & CVD

Please answer the following questions to enable the survey to be tailored to your areas of expertise and / or experience.

A1: Please select the item that best describes you.

If both are applicable, select the perspective from which you would prefer to respond in this survey.

- An expert in research, treatment, or management of diabetes and / or cardiovascular disease (e.g. a researcher, doctor)
- A person who has knowledge of the lived experience of diabetes and / or cardiovascular disease (e.g. someone who lives with one or both of these conditions, a carer or family member of someone who lives with one or both of these conditions, or a person working in an advocacy organisation)

LOGIC - show A2 if A1 = "A person who has knowledge..."

A2: What is the nature of your experience with diabetes and / or cardiovascular disease?
- I am a person living with diabetes and / or cardiovascular disease
- I am a carer or family member of a person living with diabetes and / or cardiovascular disease
- I work in the area of diabetes and / or cardiovascular disease

LOGIC - show A3 to ALL respondents
Wording of no. 3 depends on response to A1 (consumers shown plain language version)

A3: This survey contains several sections. Which of the sections listed below do you feel able to contribute your expertise and / or experience to? Tick all that apply.

1. Preventing and managing complications associated with diabetes
2. Preventing and managing complications associated with cardiovascular disease
3. SHOW IF A1 = "An expert…": Exploring interactions in the pathogenesis of Type 1 diabetes, Type 2 diabetes, and cardiovascular disease that may lead to new interventions for people with these conditions

   SHOW IF A1 = "A person who has knowledge…": The experience of [you OR those you know] living with two of the following: Type 1 diabetes, Type 2 diabetes, or cardiovascular disease

LOGIC - show A4 to ALL respondents

A4: What country do you currently reside in?

- [Dropdown list of countries]

LOGIC - show A5 if A4 = "Australia"

A5: Which state or territory do you currently reside in?

- Australian Capital Territory
- New South Wales
- Northern Territory
- Queensland
- South Australia
- Tasmania
- Victoria
- Western Australia

LOGIC - show A6 to ALL respondents

A6: Which of the following best describes where you live?

- Major city
- Regional city or town
- Rural area
- Remote area
- Other (please specify)

LOGIC - show A7 to ALL respondents

A7: Are you of Aboriginal or Torres Strait Islander origin?
(For persons of both Aboriginal and Torres Strait Islander origin, mark both 'Yes' boxes)

- No
- Yes, Aboriginal
- Yes, Torres Strait Islander
- Prefer not to answer

LOGIC - show A8 to ALL respondents

A8: Do you speak a language other than English at home?

- No, English only
- Yes, other language (please specify)
- Prefer not to say

LOGIC - show A9 to ALL respondents

A9: In which country were you born?

- Australia
- Other (please specify)
- Prefer not to say

LOGIC - show A10 if
- **A1 = "An expert..." OR**
- **A2 = "I work in the area..."**

A10: What is your profession? *Select all that apply.*
- Advocate / consumer representative for people with diabetes and / or cardiovascular disease
- Dentist
- Dietitian
- General practitioner
- Health service manager or administrator
- Medical specialist (please specify): [text]
- Nurse
- Occupational therapist
- Osteopath

- Physiotherapist
- Pharmacist
- Podiatrist
- Policy maker
- Psychologist
- Researcher
- Speech pathologist
- Social worker
- Other (please specify)

LOGIC - show A11 if
- **A1 = "An expert..." OR**
- **A2 = "I work in the area..."**

A11: What is your work setting?
- Advocacy /other organisation representing people with diabetes and / or cardiovascular disease
- Hospital
- Government
- Community
- Medical technology, biomedical or pharmaceutical industry
- Private practice
- Professional college
- University or research institute
- Other (please specify)

LOGIC - show A12 if
- **A1 = "An expert..." OR**
- **A2 = "I work in the area..."**

A12: Which of the following best describes the **primary geographical focus of** your work?
- Major city
- Regional city or town
- Rural
- Remote
- Other (please specify)
- My work does not have a geographical focus

LOGIC - show A13 if
- **A1 = "An expert..." OR**
- **A2 = "I work in the area..."**

A13: What level of engagement with Aboriginal or Torres Strait Islander communities does your

work involve?

- Minimal or no engagement with Aboriginal or Torres Strait Islander communities (i.e. not a research focus and / or less than 10% of clinical encounters)
- Moderate engagement with Aboriginal or Torres Strait Islander communities (i.e. some research focus and / or 11 - 50% of clinical encounters)
- Deep engagement with Aboriginal or Torres Strait Islander communities (i.e. research focus and / or over 50% of clinical encounters)

LOGIC - show A14 if
- **A1 = "An expert..." OR**
- **A2 = "I work in the area..."**

A14: How many years have you been in your field for?

- Less than 5 years
- 5 - 10 years
- 11 - 20 years
- 21 - 30 years
- More than 31

LOGIC - show A15 if A1 = "A person who has knowledge..."
AND NOT A2 = "I work in the area..."

A15: How many years of lived experience do you have with diabetes and / or cardiovascular disease?
If you have diabetes and / or cardiovascular disease, this means how many years you have had the disease(s) for. If you are a carer or family member, this means how many years you have been caring for someone with the disease(s).

- Less than 5 years
- 5 - 10 years
- 11 - 20 years
- 21 - 30 years
- More than 31 years

LOGIC - show A13 to ALL respondents

A16: What is your gender?

- Male
- Female
- Other (please specify)
- Prefer not to say

LOGIC - show A14 to ALL respondents

A17: What is your age in years?

- [text response - validated as number]
- Prefer not to say

SECTION B: Preventing and managing complications associated with diabetes

LOGIC - show the "SECTION B FOR EXPERTS" section if
- A1 = "An expert..." AND
- A3 = "Preventing and managing complications associated with diabetes"

SECTION B FOR EXPERTS:

This section asks about research topics for the prevention and management of complications associated with diabetes.

'Prevention and management' is broadly defined to include (but are not limited to) pharmaceutical, behavioural, device-related, diagnostic, and digital health interventions.

'Complications' refers to impacts that are not features of a disease itself, but can result from having a disease. For example, low blood sugar is a feature of diabetes; diabetic retinopathy is a complication of diabetes.

There will be one round of TTRA funding for these research topics. The focus of this funding will be on research that could have an impact on health care and patient outcomes within 4 years.

diabates_expert_intro: Please list at least two research topics that address a disease-related complication associated with diabetes where research could impact on health care and patient outcomes within 4 years.

Give your answer using the following format:

"Intervention to address **Complication** resulting in **Benefit"**

For example:

"**Remote online screening** to address **diabetic retinopathy** resulting in **less vision loss**"

"**Medication reminder systems** to address **poor adherence to medication** resulting in **lower rates of missed medication"**

"**Cheaper blood pressure measurement devices** to address **regular blood pressure monitoring** resulting in **early intervention for high blood pressure"**

diabates_expert_topic_1:

Topic 1: [INTERVENTION] to address [COMPLICATION] resulting in [BENEFIT]

- [Text response]

diabates_expert_reason_1:

Topic 1: (OPTIONAL) Why do you think this topic is important?

Your answer can refer to quality of life, healthcare or economic costs or any other impacts that this complication has.

- [OPTIONAL FREE TEXT FIELD – 50 word LIMIT]

diabates_expert_topic_2:

Topic 2: [INTERVENTION] to address [COMPLICATION] resulting in [BENEFIT]

- [Text response]

diabates_expert_reason_2:

Topic 2: (OPTIONAL) Why do you think this topic is important? Your answer can refer to quality of life, healthcare or economic costs or any other impacts that this complication has.

diabates_expert_add:

Are there any further topics you would like to list? You can list up to 8 more topics.

- Yes
- No

LOGIC - if BE_ADDITIONAL = "Yes" at the end of each loop, then

- **loop through diabates_exp_topic_3 and diabates_expert_reason_3**
- **until diabates_exp_topic_10 and diabates_expert_reason_10**

LOGIC - show the "SECTION B FOR CONSUMERS" section if

- **A1 = "A person who has knowledge..." AND**
- **A3 = "Preventing and managing complications associated with diabetes"**

'I' and 'you' wording is shown to respondents with the disease, 'those I know with diabetes' wording is shown to other consumers (e.g., carers, family members, advocates).

SECTION B FOR CONSUMERS:

This section asks about complications that result from having diabetes.

‘Complications’ refers to impacts that are not features of a disease itself, but can result from having a disease. For example, low blood sugar is a feature of diabetes; poor circulation or vision problems are complications that can result from having diabetes.

diabetes_cons_intro: What complications associated with diabetes would you like addressed by medical research, and how do they impact [you OR those you know who have diabetes]? Please list at least two complications and their impacts.

Give your answer using the following format.

“As a result of diabetes, [I OR those I know with diabetes] have [**COMPLICATION**]. This results in [**IMPACT**].

For example:

> “As a result of diabetes, I have **poor circulation**. This results in **foot infections.**”
>
> “As a result of diabetes, those I know have **nerve damage.** This results in **them not being able to feel their hands or feet very well.**”

diabetes_cons_topic_1:

As a result of diabetes, [I OR those I know with diabetes] have [COMPLICATION]. This results in [IMPACT]

diabetes_cons_topic_2:

As a result of diabetes, [I OR those I know with diabetes] have [COMPLICATION]. This results in [IMPACT]

diabetes_cons_add:

Are there any further complications and impacts you would like to list? You can list up to 8 more complications and impacts.

- Yes
- No

LOGIC - if diabetes_cons_topic_add = "Yes" at the end of each loop, then
- **loop through diabetes_cons_topic_3**
- **until diabetes_cons_topic_10**

SECTION C: Preventing and managing complications associated with cardiovascular disease

LOGIC - show the "SECTION C FOR EXPERTS" section if
- A1 = "An expert…" AND
- A3 = "Preventing and managing complications associated with cardiovascular disease"

SECTION C FOR EXPERTS:

This section asks about research topics for the prevention and management of complications associated with cardiovascular disease.

'Prevention and management' is broadly defined to include (but are not limited to) pharmaceutical, behavioural, device-related, diagnostic, and digital health interventions.

'Complications' refers to impacts that are not features of a disease itself, but can result from having a disease. For example, high blood pressure is a feature of cardiovascular disease; stroke is a complication of cardiovascular disease.

There will be one round of TTRA funding for these research topics. The focus of this funding will be on research that could have an impact on health care and patient outcomes within 4 years.

cvd_expert_topic_intro: Please list at least two research topics in the area of disease-related complications associated with cardiovascular disease in which you feel that research could have an impact on health care and patient outcomes within 4 years:

Give your answer using the following format:

"Intervention to address **Complication** resulting in **Benefit"**

For example:

> "**Aspirin** to address **blood thickening** resulting in **lowered risk of stroke**"
>
> "**Medication reminder systems** to address **poor adherence to medication** resulting in **lower rates of missed medication"**
>
> "**Cheaper devices** to addres**home blood pressure monitoring** resulting in **improved control of hypertension"**

cvd_expert_topic_1:

Topic 1: [INTERVENTION] to address [COMPLICATION] resulting in [BENEFIT]
- [Text response]

cvd_expert_reason_1:

Topic 1: (OPTIONAL) Why do you think this topic is important? Your answer can refer to quality of life, healthcare or economic costs or any other impacts that this complication has.

- [OPTIONAL FREE TEXT FIELD – 50 word LIMIT]

cvd_expert_topic_2:

Topic 2: [INTERVENTION] to address [COMPLICATION] resulting in [BENEFIT]

- [Text response]

cvd_expert_reason_2:

Topic 2: (OPTIONAL) Why do you think this topic is important? Your answer can refer to quality of life, healthcare or economic costs or any other impacts that this complication has.

- [text response - validation of 50 words]

cvd_expert_add:

Are there any further topics you would like to list? You can list up to 8 more topics.

- Yes
- No

LOGIC - if cvd_expert_add= "Yes" at the end of each loop, then
- **loop through cvd_expert_topic_3 and cvd_expert_reason_3**
- **until cvd_expert_topic_10 and cvd_expert_reason_10**

LOGIC - show the "SECTION C FOR CONSUMERS" section if
- **A1 = "A person who has knowledge..."" AND**
- **A3 = "Preventing and managing complications associated with cardiovascular disease"**

'I' and 'you' wording is shown to respondents with the disease, 'those I know with cardiovascular disease wording is shown to other consumers (e.g., carers, family members, advocates).

SECTION C FOR CONSUMERS

This section asks about complications associated with having cardiovascular disease.

'Complications' refers to impacts that are not features of a disease itself, but can result from having a disease. For example, high blood pressure is a feature of cardiovascular disease; reduced fitness due limited ability to exercise is a complication associated with cardiovascular disease.

cvd_cons_intro: What complications associated with cardiovascular disease would you like addressed by medical research, and how do they impact [you OR those I know with cardiovascular disease]? Please list at least two complications and their impacts.

Give your answer using the following format:

“As a result of cardiovascular disease, [I OR those I know with cardiovascular disease] have [**COMPLICATION**]. This results in [**IMPACT**].

For example:

> “As a result of cardiovascular disease, I have **limited exercise tolerance**. This results in **inability to walk to the shops.**”
>
> “As a result of cardiovascular disease, those I know have **vision problems.** This results in **difficulties for them when reading.**”

cvd_cons_topic_1:

As a result of cardiovascular disease, [I OR those I know with cardiovascular disease] have [COMPLICATION]. This results in [IMPACT]

cvd_cons_topic_2:

As a result of cardiovascular disease [I OR those I know with cardiovascular disease] have [COMPLICATION]. This results in [IMPACT]

cvd_cons_add:

Are there any further complications and impacts you would like to list? You can list up to 8 more complications and impacts.

- Yes
- No

LOGIC - if cvd_cons_add= "Yes" at the end of each loop, then
- loop through cvd_cons_topic_3
- until cvd_cons_topic_10

SECTION D: Exploring interactions in the pathogenesis of Type 1 diabetes, Type 2 diabetes, and cardiovascular disease that may lead to new interventions for people with these conditions

LOGIC - show the "SECTION D FOR EXPERTS" section if
- A1 = "An expert..." AND
- A3 = "**Exploring interactions in the pathogenesis of Type 1 diabetes, Type 2 diabetes, and cardiovascular disease that may lead to new interventions for people with these conditions**"

SECTION D FOR EXPERTS

This section focuses on ideas for research to better understand interactions in the pathogenesis of Type 1 diabetes, Type 2 diabetes, and cardiovascular disease that can lead to the development of interventions for people who develop any two of these three conditions.

The specific focus of these research topics is research with an identified pathway to development of an intervention that can benefit patients with two or more of these conditions.

interaction_expert_intro: Please list at least two research topics in the area of better understanding and treating interactions in the pathogenesis of Type 1 diabetes, Type 2 diabetes, and cardiovascular disease:

interaction_expert_topic_1:

Topic 1:

- [FREE TEXT FIELD – 50 word LIMIT]

interaction_expert_reason_1:

Topic 1: (OPTIONAL) Why do you think this topic is important? Your answer can refer to quality of life, healthcare or economic costs or any other impacts that this complication has.

- [OPTIONAL FREE TEXT FIELD – 50 word LIMIT]

interaction_expert_topic_2:

Topic 2:

- [FREE TEXT FIELD – 50 word LIMIT]

interaction_expert_reason_1:

Topic 2: (OPTIONAL) Why do you think this topic is important? Your answer can refer to quality of life, healthcare or economic costs or any other impacts that this complication has.

- [OPTIONAL FREE TEXT FIELD – 50 word LIMIT]

interaction_expert_add:
Are there any further research topics you would like to list? You can list up to 8 more.

- Yes
- No

LOGIC - if interaction_expert_add = "Yes" at the end of each loop, then
- **loop through interaction_expert_topic_3 and interaction_expert_reason_3**
- **until interaction_expert_topic_10 and interaction_expert_reason_10**

LOGIC - show the "SECTION D FOR CONSUMERS" section if
- A1 = "A person who has knowledge..."" AND
- A3 = "The experience of [you OR those you know living with two of the following: Type 1 diabetes, Type 2 diabetes, or cardiovascular disease"

SECTION D CONSUMERS:

This section asks about the experience of living with two both diabetes and cardiovascular disease.

When a person who experiences more than one ongoing disease, it can be difficult to know what symptoms or difficulties are due to which disease. Therefore, this question is optional - if you feel you are unable to answer the question below you do not have to.

interaction_cons_topic_1: (Optional) Is there any complication [you OR those you know] experience due to having two of the following: Type 1 diabetes, Type 2 diabetes, or cardiovascular disease, which [you OR they] didn't experience before having BOTH diseases?

- [FREE TEXT FIELD – 50 word LIMIT]

interaction_cons_impact_1: (Optional) What has been the impact of this complication?

- [FREE TEXT FIELD – 50 word LIMIT]

interaction_cons_add: Are there any further complications you would like to list? You can list up to 5 altogether.

LOGIC - if interaction_cons_add= "Yes" at the end of each loop, then
- loop through interaction_cons_topic_2 and interaction_cons_impact_2
- until interaction_cons_topic_5 and interaction_cons_impact_5

This concludes the survey, which will be submitted when you exit this page. Thank you for your contribution to this project.

Supplemental File 2: CVD Roundtable pack

Research Prioritisation Roundtable

November 25, 2020

Preventing and managing complications associated with cardiovascular disease

Participant Information Pack

Introduction

Thank you for your willingness to participate in this roundtable meeting. This information pack comprises:

1. An **agenda** for the roundtable
2. A **brief overview of the nationwide survey** that preceded this roundtable
3. A **de-duplicated list of complications** for prioritisation; and
4. **Consumer perspectives** on living with cardiovascular disease collected from the survey

Further information and instructions will be presented during the session.

Time	Agenda Activity
**12:30 – 12:50**	**Introduction**<br>- Overview of TTRA program & project, MTPConnect and project team<br>- What we are here to do, ground rules, housekeeping<br>- Briefly introduce yourself (10 seconds)<br>- ***Questions regarding process and aims of the session***<br>- Participants receive information pack
**12:50 – 12:55**	**Information pack reading time**
**12:55 – 1:15**	***Questions / clarifications regarding the information pack***<br>**Prioritisation** using the ‘Mentimeter’ platform (refer Presentation)<br>Top three topics will be identified in real time against each of the 6 criteria, and overall. Results will be presented to the group following prioritization
**1:15 – 1:25**	**[Refreshment break]**
**1:25 – 2:20**	**Breakout room discussion** of top three topic areas
**2:20 – 3:00**	**Report back**, next steps, brief roundtable survey and close

Brief overview of the nationwide survey

The survey responses which inform the topics presented at this roundtable were part of a larger survey in which 237 experts and 81 consumer responses were gathered.

Of these, 164 respondents contributed research topic ideas in the area of cardiovascular disease complications. These comprised:

- 155 experts in research, treatment, or management of cardiovascular disease - researchers (74%), medical specialists (29%) and other professions (9%). Note that respondents could select > 1 profession
- 9 consumers with knowledge of the lived experience of cardiovascular disease - people living with cardiovascular disease (55.6%) and people who worked in the area of cardiovascular disease (44.4%)

Almost all respondents were from Australia (98.8%) and respondents were equally split between females (50.0%) and males (50.0%). The state breakdown of respondents was Victoria (44.8%); New South Wales (26.4%); Queensland (10.4%); Western Australia (9.8%); South Australia (6.8%); Tasmania (1.2%); Australian Capital Territory (0.6%)

Experts were asked the following question:

> “Please list at least two research topics that address a disease-related complication associated with cardiovascular disease where research could impact on health care and patient outcomes within 4 years.
>
> Give your answer using the following format:
>
> **“Intervention** to address **Complication** resulting in **Benefit”**

A total of **300** research topics were collected and coded by **intervention** and **complication**. The list was sorted by complication (first level) and intervention (second level) and deduplicated by **complication** resulting in **19** complication categories to be prioritised at this roundtable (see page 3). Following prioritisation, discussion of complications and associated interventions will occur in two breakout rooms.

Consumers were asked the following question:

> What complications associated with cardiovascular disease would you like addressed by medical research, and how do they impact [you OR those you know who have cardiovascular disease]? Please list at least two complications and their impacts.
>
> Give your answer using the following format.
>
> “As a result of cardiovascular disease, [I OR those I know with cardiovascular disease] have [**COMPLICATION**]. This results in [**IMPACT**].”

A total of 19 complications / impacts were collected. These topics were synthesised to inform the consumer perspectives narrative in the topics table (see page 4).

De-duplicated list of complications for prioritisation

This list presents all complications described across the 300 research topics identified in the survey. Please use this list to identify your top three against the different criteria presented in the Mentimeter poll using the corresponding number. **Note that you will be using the same list for each individual criterion**.

1. Bleeding (side effect of anti-platelet therapy, post bypass)
2. Cardiac, Arrhythmia / AF
3. Cardiac, Cardiac hypertrophy
4. Cardiac, Cardiomyopathy / heart failure
5. Cardiac, Coronary artery disease / Angina / Major adverse cardiac events (MACE)
6. Cardiac, Valvular abnormalities
7. Cerebrovascular, Cognitive impairment / Dementia
8. Cerebrovascular, TIA & Stroke (Ischaemic and Haemorrhagic)
9. Complications of COVID-19
10. Effective Practice and Organisation of Care including Consumer, Adherence / knowledge; Consumer, Access to and timeliness of care, Evidence-practice gaps
11. Functional impairment including work, exercise and ADLs
12. Inflammation / Fibrosis
13. Mental illness
14. Metabolic dysregulation, glucose & lipid metabolism
15. Sleep disturbances
16. Vascular, Large vessel disease
17. Vascular, Diabetic microvascular disease / Eye, Retinopathy / Kidney disease
18. Vascular, Endocrine / secondary hypertension
19. Vascular, Peripheral vascular disease / Intermittent claudication / Ischaemic Ulcers / Amputation

Consumer perspectives

A total of 19 complications / impacts were collected. These topics were synthesised to inform the consumer perspectives narrative in the topics table below:

Complication	Consumer perspectives in relation to complication
Cerebrovascular disease; Ischaemic and haemorrhagic stroke	Three consumers reported risk of stroke as a complication. Reported issues related to this were frequent blood tests and impaired cognitive function.
Mental health	Three consumers reported mental health as a complication. Reported issues related to this were social isolation, loneliness, and depression.
Vascular complications, hypertension	Three consumers reported hypertension as a complication. Reported issues related to this were increased medication use and restricted lifestyle.
Consumer behaviour	Two consumers reported consumer motivation and adherence and a complication. This included lacking motivation and / or knowledge, and declining compliance after six months.
Lifestyle	Two consumers reported lifestyle complications including reduced exercise tolerance and inability to work.
Angina	One consumer reported chest tightness as a complication and a resulting fear of exercising.
Arrhythmia	One consumer reported atrial fibrillation as a complication.
Fatigue	One consumer reported fatigue as a complication, and difficulty exercising as a result of this.
Liver disease	One consumer reported risk of liver disease as a complication, and decreased social participation as a result of this.
Loss of function	One consumer reported general loss of function as a complication.
Musculoskeletal system, loss of muscle mass	One consumer reported loss of muscle mass as a complication.

Supplementary File 3: Diabetes Roundtable pack

Research Prioritisation Roundtable

November 24, 2020

Preventing and managing complications associated with diabetes

Participant Information Pack

Introduction

Thank you for your willingness to participate in this roundtable meeting. This information pack comprises:

1. An **agenda** for the roundtable
2. A **brief overview of the nationwide survey** that preceded this roundtable
3. A **de-duplicated list of complications** for prioritisation; and
4. **Consumer perspectives** on living with Diabetes and Cardiovascular Disease collected from the survey

Further information and instructions will be presented during the session.

Agenda

Time (AEDT)	Activity
**3:30 – 3:50pm**	**Introduction**<br>- Overview of TTRA program & project, MTPConnect and project team<br>- What we are here to do, ground rules, housekeeping<br>- Briefly introduce yourself (10 seconds)<br>- ***Questions regarding process and aims of the session***<br>- Participants receive information pack
**3:50 – 3:55pm**	**Information pack reading time**
**3:55 – 4:15pm**	***Questions / clarifications regarding the information pack***<br>**Prioritisation** using the 'Mentimeter' platform (refer Presentation)<br>Top three topics will be identified in real time against each of the 6 criteria, and overall. Results will be presented to the group following prioritization
**4:15 – 4:25pm**	**[Refreshment break]**
**4:25 – 5:20pm**	**Breakout room discussion** of top three topic areas
**5:20 – 6:00pm**	**Report back**, next steps, brief workshop survey and close

Brief overview of the nationwide survey

The survey responses which inform the topics presented at this roundtable were part of a larger survey in which 237 expert and 81 consumer responses were gathered. Of these, 166 respondents contributed research topic ideas in the area of diabetes complications. These comprised:

- 133 experts in research, treatment, or management of diabetes - researchers (74%), medical specialists (23%) and other professions (8%). Note that respondents could select > 1 profession
- 30 consumers with knowledge of the lived experience of diabetes - people living with diabetes (76.7%); people who worked in the area of diabetes (20.0%); and carers / family members of people living with diabetes (3.3%)

Almost all respondents were from Australia (98.2%), with an approximately equal split between females (48.8%) and males (51.2%). The state breakdown of respondents was Victoria (40.1%); New South Wales (28.4%); Queensland (14.8%); Western Australia (9.3%); South Australia (4.9%); Australian Capital Territory (1.8%); Tasmania (0.6%)

Experts were asked the following question:

> "Please list at least two research topics that address a disease-related complication associated with diabetes where research could impact on health care and patient outcomes within 4 years.
>
> Give your answer using the following format:
>
> **"Intervention** to address **Complication** resulting in **Benefit"**

A total of **297** research topics were collected and coded by **intervention** and **complication**. The list was sorted by complication (first level) and intervention (second level) and deduplicated by **complication** resulting in **24** complication categories to be prioritised at this round table (see page 3). Following prioritisation, discussion of complications and associated interventions will occur in two breakout rooms.

Consumers were asked the following question:

> What complications associated with diabetes would you like addressed by medical research, and how do they impact [you OR those you know who have diabetes]? Please list at least two complications and their impacts.
>
> Give your answer using the following format.
>
> "As a result of diabetes, [I OR those I know with diabetes] have [**COMPLICATION**]. This results in [**IMPACT**]."

A total of 54 complications / impacts were collected. These topics were synthesised to inform the consumer perspectives narrative in the topics table (see page 4).

De-duplicated list of complications for prioritisation

This list presents all complications described across the 297 research topics identified in the survey. Please use this list to identify your top three against the different criteria presented in the Mentimeter poll using the corresponding number. **Note that you will be using the same list for each individual criterion**.

1. Bone, Impaired osseointegration
2. Cardiac, CVD / coronary artery disease / Major adverse cardiac events (MACE)
3. Cardiac, Cardiomyopathy / heart failure
4. Consumer behaviour (adherence, knowledge)
5. Diabetic foot
6. Diabetic kidney disease (DKD)
7. Eye, Retinopathy
8. Glucose control, Hypoglycaemia, Hyperglycaemia, Ketoacidosis
9. Glucose control, Insulin resistance
10. Hepatic, Fibrosis / Non-alcoholic fatty liver disease (NAFLD) / steatohepatitis (NASH)
11. Immunosuppression
12. Mental health
13. Metabolic disturbances / Lipid disturbances
14. Myopathy (skeletal muscle)
15. Neuropathy
16. Pancreatitis
17. Periodontitis
18. Reproductive health, Gestational diabetes & consequences
19. Skin (ulcer, wound healing)
20. Vascular, Atherosclerosis / Thrombosis
21. Vascular, Hypertension
22. Vascular, Peripheral artery disease / Intermittent claudication
23. Vascular, Stroke
24. Weight gain

Consumer perspectives

A total of 54 complications / impacts were collected. These topics were synthesised to inform the consumer perspectives narrative in the topics table below:

Complication	Consumer perspectives in relation to complication
Neuropathy	Thirteen consumers reported neuropathy complications. Reported issues related to this were increased sensitivity, pain, poor circulation, and nerve damage.
Mental health	Ten consumers reported mental health as a complication. Reported issues related to this were worry about insulin doses, anxiety over eating and socialising, personality changes, emotional burnout, isolation and stigma, and depression.
Glucose control	Five consumers reported glucose control as a complication. Reported issues that arise from this were expenses, vision issues, hypoglycaemia, diabetic ketoacidosis, and fear.
Eye complications	Four consumers reported eye complications including eyesight deterioration leading to vision loss and blindness.
Musculoskeletal system, Foot complications	Four consumers reported foot complications including foot infections, loss of nerve functioning, and Charcot foot.
Skin, wound healing	Four consumers reported wound healing complications including ulcers and delays in healing small wounds and sun damage.
Cardiac complications	Three consumers reported cardiac complications including arrhythmia and heart attacks.
Renal (nephropathy)	Three consumers reported kidney damage / failure and being dependent on dialysis as a complication.
Comorbidity / interacting treatments	One consumer reported complications resulting from having other conditions and interacting medications.
Fatigue	One consumer reported fatigue as a complication.
Immunosuppression	One consumer reported dysfunctional immune system as a complication.
Instability	One consumer reported instability and difficulty walking as a complication.
Sexual dysfunction	One consumer reported sexual dysfunction as a complication.
Sleep disturbances	One consumer reported sleep disturbances as a complication.
Weight gain	One consumer reported weight gain as a complication. Reported issues related to this were impacts on joints and ability to exercise.

Supplementary File 4: Interactions Roundtable pack

Research Prioritisation Roundtable
November 23, 2020

Prioritising research topics for T1DM, T2DM and CVD interactions

Participant Information Pack

Introduction
Thank you for your willingness to participate in this roundtable meeting.

This information pack comprises:

1. An **agenda** for the roundtable
2. A **brief overview of the nationwide survey** that preceded this roundtable
3. A **de-duplicated list of complications** for prioritisation; and
4. **Consumer perspectives** on living with Diabetes and Cardiovascular Disease collected from the survey

Further information and instructions will be presented during the session.

Brief overview of the nationwide survey

The survey responses which inform the topics presented at this roundtable were part of a larger survey in which 237 experts and 81 consumer responses were gathered.

Of these, 191 respondents contributed research topic ideas in the area of the pathogenesis of Type 1 diabetes, Type 2 diabetes, and cardiovascular disease. These comprised:

- 130 experts in interactions in the pathogenesis of Type 1 diabetes, Type 2 diabetes, and cardiovascular disease that may lead to new interventions for people with these conditions - researchers (78%), medical specialists (31%) and other professions (5%). Note that respondents could select > 1 profession
- 61 consumers with knowledge of the lived experience of two or more of the following: Type 1 diabetes, Type 2 diabetes, or cardiovascular disease - people living with two or more of these conditions (73.8%); people who worked in the area (16.4%); and carers / family members of people living with two or more of these conditions (9.8%)

Almost all respondents were from Australia (97.9%), with an approximately equal split between females (47.9%) and males (52.1%). The state breakdown of respondents was:

- Victoria (36.0%)
- New South Wales (31.7%)
- Queensland (14.5%)
- Western Australia (9.1%)
- South Australia (4.3%)
- Australian Capital Territory (3.2%)
- Tasmania (1.1%)

Experts were asked to “list at least two research topics in the area of better understanding and treating interactions in the pathogenesis of Type 1 diabetes, Type 2 diabetes, and cardiovascular disease”.

A total of 209 research topics were collected and coded with respect to the **treatment, mechanism** and **complication** pertaining to each response. The list was then sorted by **complication** and deduplicated. **29 unique complication categories were identified**. These will be the unit of prioritisation at this round table.

De-duplicated list of complications for prioritisation

This list presents all complications described across the 209 research topics identified in the survey. Please use this list to identify your top three against the different criteria presented in the Mentimeter poll using the corresponding number. **Note that you will be using the same list for each individual criterion**.

1. Cardiac, Not otherwise stated (NOS)
2. Cardiac, Arrhythmia
3. Cardiac, Cardiomyopathy
4. Cardiac, CVD
5. Cardiac, Heart failure
6. Clinician and Consumer factors, Evidence-Practice gaps
7. COVID-19 mortality
8. Diabetic Kidney Disease
9. Eye, NOS
10. Eye, Retinopathy
11. Alzheimer's disease / Dementia
12. Immunosuppression
13. Metabolic disturbances
14. Sleep disturbances
15. Weight gain / weight loss
16. Glucose control, NOS
17. Glucose control, Insulin resistance
18. Hypertension
19. Inflammation (target not specified)
20. Liver, Fibrosis
21. Liver, Non-alcoholic fatty liver disease (NAFLD) and steatohepatitis (NASH)
22. Mental illness
23. Neuropathy (target not specified)
24. Primary prevention
25. Skin, Ulcer / Wound healing
26. Vascular, NOS
27. Vascular, Atherosclerosis
28. Vascular, Thombosis
29. Vascular, Ischaemia / Peripheral arterial disease (PAD)

Consumer perspectives

Consumers were asked to list any complication experienced due to having two or more of the following: Type 1 diabetes, Type 2 diabetes, or cardiovascular disease, which were not experienced before having BOTH diseases. A total of 33 complications were collected. These topics were synthesised to inform the consumer perspectives narrative in the topics table below:

**Complication**	**Consumer perspectives in relation to complication**
Mental health	Six consumers reported mental health as a complication. Reported issues related to this were depression, anxiety, stress, frustration, disappointment, and severe mood changes.
Neuropathy	Five consumers reported neuropathy as a complication. Reported issues related to this were circulation problems, inability to partake in activities, itchiness, pain, and increased hospitalisation.
Glucose control	Four consumers reported glucose control as a complication, including hypoglycaemic events. Reported issues related to this were black outs, complications after exercising, and hospitalisation.
Genitourinary system, Renal (nephropathy)	Three consumers reported kidney disease or impairment as a complication. Reported issues related to this were breathlessness, blood pressure, medication constraints, and mortality.
Weight gain	Three consumers reported weight gain as a complication. Reported issues related to this were impacts on fitness, joints, and mental health.
Knowledge	Two consumers reported confusion with regards to correct medication.
Angina	One consumer reported angina as a complication, particular while exercising.
Gastrointestinal system, bowel	One consumer reported bowel issues as complications. Reported issues related to this were pain and inconvenience.
Genitourinary system, sexual dysfunction	One consumer reported impotence as a complication.
Fatigue	One consumer reported fatigue as a complication, and difficulty exercising / increased weight as a result of this.
Lifestyle	One consumer reported lifestyle complications including lower educational attainment for teenagers.
Sleep disturbances	One consumer reported sleep disturbances as a complication that resulted in affected mood and ability to exercise.
Treatment side effects	One consumer reported side effects of treatments as a complication that resulted in weight gain.

Supplementary File 5: Survey Demographics

Demographics	Survey section			
	CVD (n = 164)	Diabetes (n = 163)	Interactions (n = 191)	Total (n = 318)
**Gender**				
Male	50%	52%	52%	49%
Female	50%	48%	48%	51%
**Role**				
Experts	95%	82%	68.1%	75%
Consumers	5%	18%	31.9%	25%
**State**				
Australian Capital Territory	1%	2%	3%	2%
New South Wales	27%	29%	32%	29%
Northern Territory	0%	0%	0%	0%
Queensland	11%	15%	15%	15%
South Australia	7%	5%	4%	6%
Tasmania	1%	1%	1%	1%
Victoria	44%	40%	36%	38%
Western Australia	10%	9%	9%	9%
**Primary work focus**				
Major city	63%	70%	68%	65%
Regional city or town	9%	6%	9%	8%
Remote	1%	1%	0%	0%
Rural	1%	1%	1%	1%
No geographical focus	20%	19%	17%	21%
Other	6%	4%	4%	5%
**Engagement with Aboriginal and Torres Strait Islander communities**				
Minimal or no engagement	67%	65%	72%	72%
Moderate engagement	31%	33%	25%	26%
Deep engagement	3%	2%	2%	2%
